# Short stature related to Growth Hormone Insensitivity (GHI) in childhood

**DOI:** 10.3389/fendo.2023.1141039

**Published:** 2023-03-15

**Authors:** Concetta Mastromauro, Cosimo Giannini, Francesco Chiarelli

**Affiliations:** ^1^ Department of Pediatrics, University of Chieti, Chieti, Italy; ^2^ Center of Advanced Studies and Technology – CAST (ex CesSI-MeT), University of Chieti, Chieti, Italy

**Keywords:** short stature, GH receptor, GH insensitivity, growth hormone, GH-IGF-1 axis, childhood

## Abstract

Linear growth during childhood is the result of the synergic contribution of different factors. The best growth determinant system during each period of life is represented by the growth hormone–insulin-like growth factor axis (GH–IGF), even if several other factors are involved in normal growth. Within the broad spectrum of growth disorders, an increased importance has been placed on growth hormone insensitivity (GHI). GHI was reported for the first time by Laron as a syndrome characterized by short stature due to GH receptor (GHR) mutation. To date, it is recognized that GHI represents a wide diagnostic category, including a broad spectrum of defects. The peculiar characteristic of GHI is the low IGF-1 levels associated with normal or elevated GH levels and the lack of IGF-1 response after GH administration. Recombinant IGF-1 preparations may be used in the treatment of these patients.

## 1 Introduction

Linear growth during childhood is a complex process regulated by both prenatal factors and nutritional, hormonal, environmental, or genetic components, the latter subject to increasing importance. In fact, it is known that adult height is an inheritable trait and results from the synergic contribution of each polymorphism among the genes associated with linear growth. It has been postulated that every single abnormality of these genes could significantly impair linear growth during childhood, and this is related to short stature (1). However, only a small proportion of these genes is recognized to be related to growth disorders during childhood. The newest genetic techniques will probably allow the detection of a rising number of gene mutations, which will explain the underlying cause of short stature.

Within the broad spectrum of growth disorders, an increased importance has been placed on growth hormone insensitivity (GHI). GHI was first described by Laron in 1966. For many years, GHI and Laron syndrome were considered the same entity, and the phenotype characterizing Laron syndrome was the only one recognized among the GHI syndrome. Particularly, it was known that GHI is first caused by a defect in the GH receptor (GHR). In fact, the mutations of GHR lead to impaired binding of GH to GHR and consequently to the lack of IGF-1 production that is secreted after GHR activation. With the development of molecular techniques that allow the cloning and characterization of the human GHR, the pathophysiology of GHI has been better understood (2, 3). The discovery of novel genes related to growth has allowed a more complete study of genetic abnormalities in the GH–IGF-1 axis, thus providing a better understanding of the complexity of GHI and the physiology of human linear growth. To date, it is recognized that GHI represents a wide diagnostic category, including a broad spectrum of defects affecting the function of the IGF-1 system. These abnormalities may involve gene coding for proteins, both those controlling GH binding or signal transduction and IGF-1 synthesis, transport, or action, and are associated with a variety of phenotypes and biochemical abnormalities (3). The common characteristics of all these defects are represented by short stature, which may be associated with peculiar characteristics specific to each defect, although in the majority of cases the genotype–phenotype correlation is not yet clarified.

The purpose of this review is to describe the common genetic abnormalities related to the spectrum of GHI, focusing on those determining IGF-1 deficiency in order to better clarify each single entity of this complex group and to promote a tailored approach based on genetic features.

## 2 Physiology of normal growth

Short stature in children is defined as a height standard deviation score (SDS) lower than - 2 SD, more than 2 SD below a population’s mean for age and sex (4). Although this definition is well recognized, it is important to state that intrauterine period, infancy, childhood, and adolescence are characterized by different growth patterns that might affect growth differently. In particular, intrauterine growth is mainly influenced by maternal nutrition, placental factors and the intrauterine milieu. During this period, the genetic influence seems to have less importance, as demonstrated by the poor correlation between birth length and adult height (5). The first two years of life are initially characterized by a rapid growth, followed by a successive deceleration. During this period, the child’s genetic potential and endocrine factors start to be expressed. Childhood age is characterized by a constant linear growth that rapidly increases soon after the pubertal period (6). However, despite the different factors influencing each period of life, the best growth determinant system at all ages remains the GH–IGF-1 axis, even if several other factors are involved in normal growth (7).

### 2.1 GH-IGF-1 axis

Regular GH-IGF-1 secretion and functions are fundamental for pre and postnatal growth (3). Human prenatal growth is mainly regulated by nutritional supplies, which influence fetal IGF-1 and, perhaps, IGF-2 (8).

**Figure 1:**
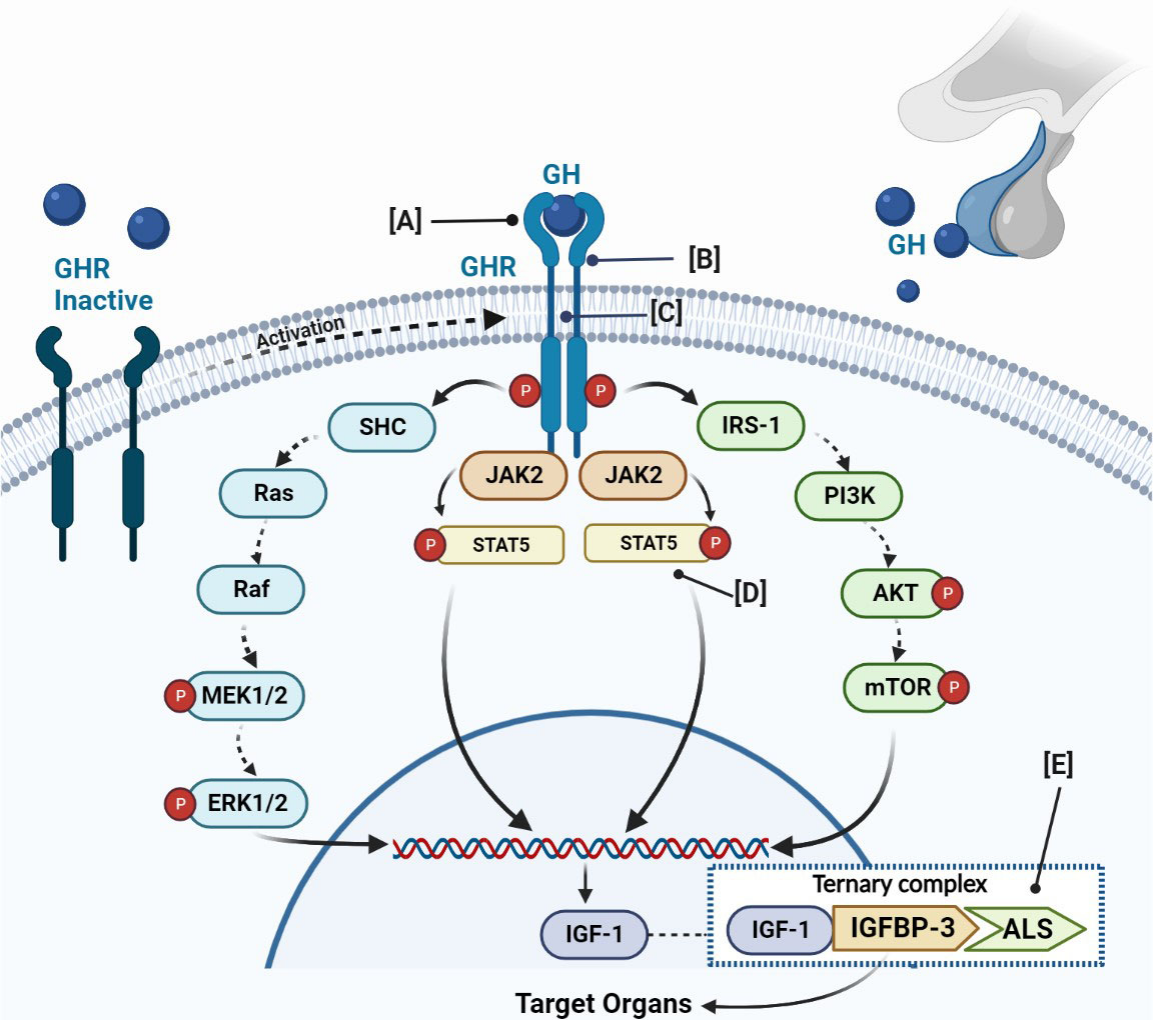
Schematic figure of GH-IGF-1 axis, its physiology, and main genetic defects related to GHI. The GH binds to a GHR, which activates JAK2 and promotes the phosphorylation of different members of the signal transducer. The final result is the IGF-1 production. IGF-1 binds to IGFBP-3 or IGFBP-5 and the acid-labile subunit (ALS) and constitutes the ternary complex. **(A)** Defects of the extracellular domain of the GHR; **(B)** defects in GHR dimerization; **(C)** defects of the transmembrane domain of the GHR; **(D)** defects of STAT5b; **(E)** defects of IGFBP. GH, growth hormone; GHR, growth hormone receptor; JAK2, Janus family tyrosine kinase 2; PI3K, phosphatidylinositol-3 kinase; ERK, extracellular signal-regulated kinase; STAT, signal transducer and activator of transcription; IGF, insulin-like growth factor; IGFBP, IGF-binding proteins.; ALS, acid-labile subunit.

Pituitary GH is encoded by the GH1 gene, and it is secreted in pulsatile manner in the circulation (9). GH production is modulated by neurological, metabolic, and endocrine factors. Various hormonal stimuli, both stimulatory—such as hypothalamic GH-releasing hormone (GHRH), ghrelin, and sex steroids—and inhibitory—such as somatostatin, IGF-1, and glucocorticoids—regulate this balance. GH exists free of or alternatively bound to the GH-binding protein (GHBP), which is a portion of the receptor, thus constituting half of the total GH amount. After secretion, GH binds to a specific receptor, namely, growth hormone receptor (GHR), which is mainly expressed in the liver, bone, muscle, and other target tissues (**Figure 1**). In turn, after receptor occupation and dimerization, the intracellular effects start. The final result of this complex chain of events is the synthesis of IGF-1 and IGF-2, which through endocrine, paracrine, and autocrine mechanisms stimulates linear growth (10). In particular, IGF-2 and IGF-1 are the major effectors of growth during fetal life, while IGF-1 is produced throughout life. Studies performed on mice have demonstrated that tailored disruption of either IGF-1 or IGF-2 led to a 40% decrease in fetal growth (8). On the other hand, GH and the GH-IGF-1 axis are the major protagonists of human postnatal growth, which may be impaired by mutations affecting every level of this complex axis.

In addition, GH can act in a direct manner despite the indirect actions through the IGF-1 release (7). In fact, the original concept, namely, the ‘‘somatomedin hypothesis’’, postulates that after GH binds to its receptor, IGF-1 is produced and independently influences growth (3, 11). This concept has evolved over the last years, and Green and colleagues proposed the “dual effector hypothesis”, postulating that GH regulates the expression of locally produced IGF-1, which then acts in an autocrine/paracrine manner (12). In addition, different studies have suggested local effects of GH that are independent of those mediated by circulating IGF-1. This hypothesis has been confirmed by Isaksson and others who demonstrated that GH stimulates the differentiation of preadipocytes and chondrocytes in the growth plate, while IGF-1 stimulates their clonal expansion (13, 14).

Thereafter, IGF-1 is primarily secreted into the blood and then links to one of six high-affinity IGF-1 binding proteins (IGFBPs). For 75–90%, IGF-1 is linked to IGFBP-3, which is the most abundant among the IGFBPs family, and for 1%, circulates unbound. The linkage between IGF-1 and IGFBP-3 constitutes a complex called the “binary complex”. Soon after, this binary complex is stabilized by the linking to an acid-labile subunit (ALS), codified by the gene IGFALS into the liver. The ternary complex reduces IGF-1 and IGF-2 circulating levels and increases their half-life (15). In turn, IGF-1 binds to a membrane-spanning homodimeric receptor (IGF-1R), which determines the autophosphorylation of the intracellular β-subunit of IGF-1R and the stimulation of intracellular signaling pathways (16).

### 2.2 Growth hormone receptor (GHR)

The GHR mediates the effects of GH on linear growth and metabolism. It is ubiquitously expressed with major concentrations in the liver. The GHR gene is sited on chromosome 5p13-p12 (17). It is a protein composed of 620 amino acids, including an extracellular, transmembrane, and intracellular domain. The binding extracellular domain of 246 amino acids is involved in GH binding through the subdomain 1 and in GHR dimerization across the subdomain 2. The single transmembrane domain is composed of 24 amino acids that anchor the receptor to the cell surface. Finally, the intracellular domain of 350 amino acids is fundamental for GH signaling (18). The receptor is encoded by nine exons, namely from 2 to 10 (19). GH binding to its receptor determines receptor activation through rotation, the changes of the conformation and dimerization thus constituting a ternary complex between two GHR and one GH molecule (20). Thereafter, a cleavage of the GHR determines the release of its extracellular domain, which circulates in blood as a soluble GH-binding protein (GHBP). The boxes 1 and 2, located in the intracellular domain of the GHR, are important for the GHR-GH–IGF-1 axis signaling transduction, since they contain the JAK2 binding sites, which are linked and activated by Janus Kinase 2 (JAK2) (**Figure 1**). The linkage of JAK2 to GHR is fundamental for controlling the position of the GHR transmembrane helices, its movements, and the crystal structures of the JAK2 kinase. On the other hand, recent studies have revealed that the GH receptor may exist as constitutive dimers rather than being dimerized as a consequence of ligand binding. Binding of the bivalent ligand reorientates and rotates the receptor subunits, determining the conversion from a form with parallel transmembrane domain to one where the transmembrane domain is divided at the point of entry into the cytoplasm (21). This movement slides the pseudokinase inhibitory domain of one JAK kinase away from the kinase domain of the other JAK, allowing the two kinase domains to interact and transactivate (21). This determines the tyrosine phosphorylation within the receptor cytoplasmic domain, which generates docking sites for the SH2 domain that contains proteins such as STAT3 or STAT5, which are phosphorylated and activated (22). Receptor phosphorylation is accompanied by the direct JAK2 phosphorylation of other target proteins (21).

Thus, JAK2 represents a potential modifier of signaling, either by inhibiting activation or activating the receptor in the absence of the ligand. In addition, it has the advantage of activating a receptor that is insensitive to the ligand due to mutations in ligand-binding residues (21).

However, to promote GH action, in addition to this pathway namely Janus kinase-signal transducers and activators of transcription (JAK-STAT), GH signal transduction is mediated by other two pathways: phosphatidylinositol-3 kinase (PI3K) and Mitogen-activated protein kinase (MAPK). The final result of the activation of these signals is represented by IGF-1 production. The normal functions of GHR are essential to ensure the physiological effects of GH (23) not only in terms of linear growth but also in terms of bone mineral density and adiposity, with a greater risk of health consequences like osteoporosis, lipid disorders, and cardiovascular diseases (22).

## 3 Growth hormone insensitivity (GHI)

Resistance or insensitivity to hormone action is defined when normal concentrations of a specific hormone are unable to induce the usual response; thus, the secretion of the proximal hormone is increased. In detail, GH insensitivity (GHI) is characterized by low IGF-1 levels associated with normal or elevated GH levels and lack of IGF-1 response after GH administration (24). The pathognomonic biochemical feature of the different entity of GHI is represented by IGF-1 deficiency (25).

Several genetic defects are responsible for the impairment of GH and IGF-1 actions, resulting in short stature that could be manifest during both the prenatal and postnatal period. GHI was reported for the first time by Laron and colleagues in two siblings with the classical clinical appearance of GH deficiency, but presenting elevated levels of GH (26). To date, the spectrum of GH insensitivity has been considerably expanded thanks to advances in terms of genetic diagnosis, leading to the discovery of different mutations affecting every level of the GH-IGF-1 axis (3).

GH insensitivity can be classified as related to primary GH insensitivity, associated with IGF-1 deficiency and IGF-1 insensitivity, and secondary GH insensitivity. The GH insensitivity resulting from IGF-1 deficiency can be categorized into different groups, namely, defects of the GH receptor (GHR), defects of the intracellular GH signaling pathway (STAT5B), and primary defects of the synthesis or activity of IGF-1 and IGF-2 (27), as shown in **Table 1**.

**Table 1:** The main defects associated with GH insensitivity in children and their clinical and laboratory features.

	DEFECTS	CLINICAL FEATURES	LABORATORY FEATURES
**PRIMARY** **GH** **INSENSITIVITY**	GHR - Extracellular domain - Transmembrane - Intracellular domain - Exon	- Severe/mild short stature - Facial hypoplasia - Obesity - Insulin resistance/glucose intolerance/Type 2 diabetes	GH **↑,** IGF-1 **↓,** IGFBP-3 N**/↓,** variable GHBP
	Intracellular GH signaling pathway (STAT5B, STAT3)	- Severe short stature - Immune deficiency	GH↑, IGF-1 ↓↓, IGFBP-3 N/↓↓, GHBP N
	IGF1/IGF2 synthesis	- Short stature - Deafness - Intellectual disability - Microcephaly - Carbohydrate intolerance	GH↑, IGF-1 ↓↓ or absent, IGFBP-3 N, GHBP N
	IGF-1 receptor deficiency/Bioinactive IGF-1	- Severe intrauterine growth restriction - CNS abnormalities	GH↑, IGF-1 ↓↓ or absent, IGFBP-3 N, GHBP N
	IGFBP-3/ALS	- Mild short stature - Delayed puberty - Insulin insensitivity - Decreased bone mineral density	GH↑, IGF-1 ↓, IGFBP3 **↓↓,** GHBP N
**SECONDARY** **GH** **INSENSITIVITY**	- Antibodies against GH - Antibodies against GHR - Malnutrition - Inflammatory bowel diseases - Severe disease - Catabolic state - Liver diseases - Poorly controlled diabetes	- Variable short stature - Typical features characterizing the underlying disease	GH **↑,** variable IGF-1, GHBP **↓,** IGFBP-3 ↓/N

### 3.1 Primary GH insensitivity

#### 3.1.1 Growth disorders related to GHR defects

Mutations of GHR represent the most frequent cause of primary GHI syndrome, clinically characterized by severe short stature, with a height up to 10 standard deviations (SDs) below normal, and severe IGF-1 deficiency (28). To date, over 90 different mutations of the GHR gene have been characterized (29). There are various types of mutations, including deletion, RNA-processing defects, translation, and missense mutations, which may affect every step essential for the correct functioning of the pathway, from ligand binding to signal transduction, and which finally determine the failure to stimulate normal growth (22). In each coding exon of GHR, at least one molecular abnormality has been reported, but a poor genotype–phenotype relationship has been demonstrated.

The primary and best-known hormone insensitivity syndrome is known as Laron syndrome. It was described for the first time in 1966 in two siblings among consanguineous Jewish families from Yemen (26). Only after twenty years in 1989 was the cause of the condition identified in the partial deletion of the GHR gene (30). Laron syndrome is a fully penetrant autosomal recessive disease caused by exon deletion (31) or mutations of the GHR (32), leading to the disruption or alteration of the GH-binding site or failure to express the GHR on the cell surface (**Figure 1A**). Only homozygous and double heterozygous patients for these defects express the typical phenotype. It was characterized as a clinical entity indistinguishable from congenital GH deficiency (GHD). The typical features include severe short stature, dwarfism, obesity, small genitalia, and delayed puberty (33–35). The short stature is severe, with final height ranging from -5 to -10 SDS below a population's mean for age and sex. One of the characteristic features is facial hypoplasia, which has different degrees of severity and is due to underdeveloped facial bones. The obesity starts in childhood and is characterized by high body fat localized in the arms; it is enhanced by insulin resistance that may lead to the development of glucose intolerance and type 2 diabetes (36, 37). In addition, obesity seems to be correlated to leptin levels, which are elevated in patients with homozygous GHI, probably resulting from abnormalities of the body composition and metabolism (15, 38, 39). The laboratoristic features include increased serum GH and low serum IGF-1 that does not increase after rhGH (recombinant human GH) administration.

The vast majority of the recognized molecular defects included in this category are associated with severe GHI and are usually localized in the extracellular domain of the GHR. In particular, among the 93 identified mutations of human GHR, the majority of cases—68 to be exact—are related to the extracellular domain; 13 occur in GHR introns, 10 occur in the intracellular domain, and only 2 occur in the transmembrane domain. The mutation that decrease GH binding are generally associated with the reduction of GHBP, which represents the circulating extracellular domain of the GHR, and it is decreased or undetectable in 80% of cases. The dosage of GHBP may be used as a laboratoristic parameter to differentiate the different mutations of GHR. In fact, mutations affecting the transmembrane or intracellular domains of the GHR are commonly characterized by normal, or even increased, serum concentrations of GHBP.

In addition, among the mutations affecting the extracellular domain, some might preserve GH binding even if they alter GH actions through different mechanisms (40).

The mutations occurring in the subdomain that implicate in the dimerization of GHR should be considered (**Figure 1B**). Although they preserve GH binding, different mutations are responsible for inability to form a stable GHR dimer, causing a defect of the entire system. To date, the discovered mutations affecting this function are three. The first is E180X (GAA > TAA), which results in a receptor protein with an 8-amino acid deletion in the extracellular dimerization domain. This mutant protein preserves the capacity to homodimerize, but the transferring to the cell surface is abnormal (41). The second is E180 splice, which regards both GH binding and GHR trafficking, producing a non-functional GHR (42). Finally, a deletion of 166 bases of exon 7 in the GHR mRNA was found in a patient affected by neurofibromatosis and concomitant short stature. This mutation resulted in the premature termination of the sequence and thus in a reduced GH-binding affinity to the GHR, hence determining growth failure (43).

However, there are few cases where heterozygous GHR mutations also exert a dominant negative effect (28, 44). These subjects usually present a lesser growth restriction and a milder clinical phenotype (45).

In addition, mutations of the splicing sites result in the improper translation of transcripts into biologically active proteins. Among the defects causing abnormalities of GHR splicing, an intronic base change leading to the activation of a pseudoexon sequence and an insertion of 36 new amino acids within the receptor extracellular domain were first reported in a case of GHI from a consanguineous Pakistani family (46). These defects have been shown to lead to an impaired function of the abnormal protein (47). The phenotypes are variable, from severe to mild short stature (48), since the splice site mutations form heterodimers with the normal GHR and exert a dominant-negative effect on the normal protein (3). A recent study has identified a novel GHR 6Ω pseudoexon inclusion resulting in the loss of GHR function associated with the severe GHI phenotype. This represents a novel mechanism of Laron syndrome and is the first deep intronic variant identified that is related to severe postnatal growth failure (49).

#### 3.1.2 GH signaling defects

The linking of GH to the GHR stimulates signaling cascades, involving different pathways that finally promote GH action, after the phosphorylation of different transduction factors. Among the different pathways, JAK-STAT seems to have a key role in the growth mechanisms, acting after phosphorylation of the GHR. In turn, the receptor phosphorylation promotes STATs phosphorylation and dissociation from the GHR as well as the dimerization and translocation to the nucleus, where it activates specific transcription elements on DNA and regulates the transcription of different genes, including IGF-1 (**Figure 1**). Although abnormalities in the MAPK pathway and NF-KB pathway may also cause GHI (50), the STATs mutations are the most studied correlation (**Figure 1D**). Several STATs have been identified, namely, from STATs-1 to STATs-6. All STATs have the same structure composed of five domains, including: an amino-terminus domain, fundamental for nuclear translocation and DNA binding; a coiled-coil domain; a DNA-binding domain; a SH2 domain, needed for receptor-specific recruitment and STAT dimerization; and a COOH-terminal transcriptional activation domain (19). To date, pathophysiological genetic defects have been identified in all STATs except STAT5A (51–53), which is most closely related to STAT5B, sharing >90% amino acid identity (54).

Although the GH signal transduction is mediated in part through STAT1 and STAT3, it seems that the germline STAT5B deficiency is more strongly associated with growth failure due to IGF-1 deficiency. In fact, in a study using mice knockout models (STAT5a–/–, STAT5b–/–, and STAT5a–/–b–/–), it was shown that the mice required STAT5b for the production of IGF-1 after GH treatment and to ensure normal postnatal growth (55). These results were confirmed by studies demonstrating that human STAT5B mutations also cause severe growth failure due to GHI and also demonstrating the critical role exerted by STAT5b signaling in GH-induced IGF-1 production and in normal linear growth (56). The first identification of STAT5B mutations was documented in 2003 in an Argentine 16-years old female born in a consanguineous family and affected by the homozygous missense mutation of the STAT5b gene, which determined the replacement of proline with alanine in the SH2 domain (57). The resultant protein is unable to phosphorylate normal effectors after GH stimulus (58). This patient showed severe short stature with a height lower than 10 SD for age and sex. The biochemical profile is characterized by normal or elevated GH secretion, severe IGF-1, IGFBP-3, and ALS deficiency, which does not increase after GH administration (3). On the other hand, serum GHBP values are normal in the majority of cases, reflecting the normality of the GHR gene and the produced protein (55). These defects do not affect prenatal growth, as confirmed by the normal birth weight presented by these patients. In contrast, they are characterized by severe postnatal growth failure that does not respond to exogen GH administration (58, 59).

Since the first report, about six other cases of STAT5B mutations have been documented and have been mainly demonstrated in siblings (60). All reported mutations were homozygous and autosomal recessive. With the exception of the first case, a common feature recognized in the majority of cases is immune dysfunction. As a consequence of the immunological abnormalities, the patients were affected by recurrent pulmonary infections occurring since infancy, including episodes of lymphoid interstitial pneumonia and consequently autoimmune disease (58). Other life-threatening infections include chronic diarrhea, severe eczema, herpes keratitis, herpes zoster, severe varicella, and juvenile arthritis (15, 56). However, these patients have normal brain development and cognitive functions.

As mentioned above, it is known that GH activates STAT1 and STAT3, which may regulate genes associated with growth and mediate their metabolic effects (61, 62). An essential role of STAT3 acting through IGF-1 in embryonic and perinatal growth was established (63). Mice lacking one allele of STAT3 showed more perinatal mortality, lower serum IGF-1 levels, and lower birth weight in 10–15% of cases (64). Germline heterozygous STAT3 gain of function mutations result in a heterogeneous phenotype that includes early-onset multiorgan autoimmunity, immunodeficiency, and short stature (64). In addition, the defect is associated with intrauterine growth restriction, delayed puberty, tooth eruption, and sometimes with delayed bone age. Although short stature represents the major clinical feature in these patients, in the majority of cases of STAT3 mutation, only poor data about growth are available. The common laboratoristic characteristic of all cases consists of IGF-1 deficiency associated with normal GH secretion. The molecular mechanism underlying the growth impairment is not completely understood. However, different studies have postulated the influence of chronic disease and immunosuppressive medications on growth failure (65). In addition, some STAT3 mutations have been shown to decrease STAT5B transcriptional activity, suggesting a negative impact in the GH signaling pathway (64).

#### 3.1.3 Primary defects of the synthesis or activity of IGF-1 and IGF-2

This category of defects includes defects of the synthesis of IGF-1 and IGF-2 due to gene mutation, abnormalities of the IGF-1 receptor, bio inactive IGF-1, and defects of IGFBP-3 or ALS.

The first case of growth failure due to the IGF-1 gene defect was described by Woods and colleagues in 1996 (66). It is caused by the homozygous deletion of exons 4 and 5 of the IGF-1 gene. The patients showed both intrauterine and postnatal growth failure, suggesting a key role of IGF-1 in intrauterine growth that seems to be GH-independent (19). The common features of IGF-1 deficiency include severe short stature with a height lower than -3 SDS for age and sex, high GH levels, and extremely low levels of IGF-1, normal IGFBP-3 levels, and slightly delayed bone age (15). At the clinical examination, all the cases with a homozygous IGF-1 deficiency are short and characterized by microcephaly, intellectual development delay, and sensory-neural deafness (67, 68). The microcephaly characterizing these patients allows to distinguish these defects from the others previously described. At the laboratory analysis, the cases may present variable serum IGF-1 levels and particularly those subjects with a mild phenotype present IGF-1 levels that are not extremely decreased (50). However, all the patients have normal or elevated serum IGFBP-3 and ALS levels.

The defects of the IGF-1 receptor determine GHI and severe intrauterine and sensorineural deafness. However, poor studies have been performed in humans. Similarly, the production of bioinactive IGF−1 may cause the same features of the IGF-1 receptor defects and is related to both intrauterine and postnatal growth failure (69).

On the other hand, the bioavailability of IGF-1 is dependent on its release from associated IGFBPs, which have a higher affinity for IGF-1 than IGF1R. IGFBPs thus act both as carriers of IGF, prolonging its half-life, and as modulators of IGF availability and activity (70). No human mutations in IGFBPs have been identified to date (54), but IGFALS may affect IGF-1 bioavailability. In fact, the acid−labile subunit (ALS) deficiency that is needed to form the ternary complex with IGF-1, IGFBP-3, or IGFBP-5 is responsible for IGF-1 deficiency (**Figure 1E**). However, growth is not extremely compromised in these cases. In fact, serum IGFBP-3 levels are more significantly reduced compared to those of IGF-1. In addition to common features characterizing the IGF-1 deficiency defects, the peculiar characteristic of this defect is represented by the delayed onset of puberty associated with insulin insensitivity and decreased bone mineral density (71). The insulin sensitivity is probably related to the increased GH levels and the low IGF-1 levels (72–74). The poor pubertal growth suggests that the circulating IGF-1 pool, in addition to IGF-1 produced at the growth cartilage, is essential to achieve a normal pubertal spurt (75). This reflects the clinical observations of a lack of growth acceleration during puberty in patients with IGF-1 deletion or resistance where no significant increase in circulating IGF-1 levels occurred. However, normal pubertal growth has been reported for several patients despite persistent low circulating IGF-1 levels (76). Therefore, another possible explanation for the discrepancy in the pubertal growth registered in different studies might be the low estrogen levels detected in these patients, since estrogen plays a direct role in the pubertal spurt. Since IGF-1 is involved in sensitizing the gonads to the action of the gonadotropins, the low estrogen levels might be, in turn, related to the low IGF-1 concentrations (75, 77).

### 3.2 Secondary GH resistance

In patients treated with rhGH, an immunological reaction may lead to the production of the rhGH antibody that neutralizes GH molecules. However, after ending rhGH treatment or changing the composition of preparation, the GH antibodies rapidly decrease until they disappear in most patients (78, 79). Among the secondary conditions associated with GHR resistance, the antibody that acts directly against GHR is one of the best known (15). The antibodies are directed in most cases against the extracellular domain and binding protein. The different antibodies may have several effects on the receptor, as an antagonist or agonist (80, 81). Other conditions, such as malnutrition, inflammatory bowel diseases, and chronic diseases, are associated with GH resistance (82). The resulting growth failure may vary from mild to moderate. The laboratory findings that characterize secondary GH resistance are increased GH, variable IGF-1 concentration, low GHBP, and low or normal IGFBP-3 levels (83). Another condition is hepatic resistance in children affected by poor-controlled type 1 diabetes (T1D), which is treated with insulin, resulting in the decrease of IGF-1 levels. In fact, the lack of the negative feedback exerted by IGF-1 causes GH hyper secretion. IGFBP-3 levels increase and GHBP decreases. The results are growth failure varying from mild to severe form. The severe form of growth failure in children with T1D is also called Mauriac syndrome, which is characterized by hepatomegaly and cushingoid features in addition to short stature (84). In these cases, adequate insulin therapy may reverse growth failure and hepatomegaly when present (84).

## 4 Therapy

The categorization of the defects is important to establish the correct therapeutical strategy. Different studies have demonstrated that high doses of recombinant human GH (rhGH) allow to obtain a mild increase of IGF-1 concentration for a short period. However, after the failure of the compensatory mechanism, IGF-1 production decreases despite treatment and becomes insufficient to assure normal growth, prevent delayed bone age, and affect final height (22).

Alternatively, a combined therapy comprising rhGH plus recombinant human IGF-I (rhIGF-1) appears to be an effective treatment option in some cases. In particular, it may be effective in cases characterized by the presence of heterozygous mutations in the GHR intracellular domain that have mild phenotypes compared with those of the classical GHI syndrome (28). Thus, this therapeutical approach may be useful in cases of less severe GH insensitivity, while in conditions of complete GH insensitivity the rhIGF-1 represents the only therapeutical option to improve linear growth (85).

The treatment with rhIGF-1 is indicated in cases of Laron syndrome and likewise in the mutations of the GH–GHR activation pathway and in the presence of the GH-inhibiting antibody (86). This therapy improves stature by increasing the annual height rate and has a positive effect on dysmorphic facial features typical of patients affected by Laron syndrome. The response to treatment may be more evident in patients with a more severe form of disease. Despite the acceleration of the growth rate, the final height still remains below the third percentile in the majority of cases. However, it has been demonstrated that if the therapy is started early during childhood, a near-normal adult height can be achieved. The adverse effects of the rhIGF-1 therapy include intracranial hypertension occurring in the 5% of cases, headache and transient papilledema, and lipohypertrophy and pain at the injection site (15). One of the most fearsome adverse effects is hypoglycemia, which has been described in 8% of the treated patients (87).

Therefore, it is clear that there are different therapeutical approaches based on the severity of the defect that need to be tailored according to the specific genetic mutation.

## 5 Conclusions

The GH–IGF-1 axis in humans is fundamental for normal pre and postnatal growth. The mutations at every level of this complex mechanism may result in growth impairment and consequently short stature. GHI was first described by Laron, but following the development of novel and more sophisticated molecular techniques, the pathophysiology of GHI has been better understood. To date, GHI is known to include a wide range of defects, each one with its own clinical and biochemical features that are distinct from one another. However, the exact mechanisms underlying short stature remain unknown in many patients, and the thorough assessment of patients with growth failure should be promoted in order to improve diagnosis and particularly to personalize the correct therapeutical approach.

## Author contributions

CG and CM wrote the draft. CG and FC revised the text. All authors contributed to the article and approved the submitted version.
